# Potential use of optical coherence tomography in oral potentially malignant disorders: in-vivo case series study

**DOI:** 10.1186/s12903-023-03263-w

**Published:** 2023-08-04

**Authors:** Alessio Gambino, Eugenio Martina, Vera Panzarella, Tiziana Ruggiero, Giorgia El Haddad, Roberto Broccoletti, Paolo G. Arduino

**Affiliations:** 1 Department of Surgical Sciences, CIR Dental School, University of Turin, Via Nizzan.230, 10123 Turin, Italy; 2 Department of Surgical, Oncological and Oral Sciences, University of Palermo, Palermo, Italy

**Keywords:** Optical coherence tomography, Oral soft tissue, Oral cancer, Oral potential malignant disorders

## Abstract

**Background:**

Evidence confirms that the use of Optical Coherence Tomography (OCT) in oral medicine can be a reliable aid for the diagnosis and management of Oral Potentially Malignant Disorders (OPMDs). Several authors described the ability of this system to detect the structural changes of the epithelia involved by the OPMDs. The purpose of this case series is to provide a suggestion for interpretation of OCT images from different OPMDs, compared to OCT images of healthy tissues.

**Methods:**

A sample of 11 OPMDs patients was recruited and analyzed with OCT. The images obtained were then compared with an OCT repertoire image. In this work the reflectance degree was considered, together with the analysis of the increased/decreased thicknesses of the various layers. Keratin Layer (KL), Epithelial Layer (EP), Lamina Propria (LP), Basal Membrane (BM) assessment, for each lesion, was performed.

**Results:**

OCT measurements of KL, EP and LP layers, together with BM assessing, should aid the physicians to recognize and describe different oral lesions, relating them to the corresponding oral pathology.

**Conclusion:**

More studies like this, on larger samples, are needed to validate the results and provide, in the future, a kind of manual that could guide clinicians to correctly interpret the OCT images in relation to the causing pathologies.

## Background

To date, oral cancer represents the sixth most widespread oncological pathology in the world, with an incidence of approximately 300,000 new cases diagnosed every year [1]. Oral cavity tumors account for 30% of head/neck neoplasms; of these, over 90% are squamous cell carcinomas. The 5-years survival rate is around 50% and it has not undergone particular variations in recent decades [2]. Oral cancers result from a documentable preclinical phase of potentially malignant lesions. Oral Potential Malignant Disorders (OPMDs) containing dysplasia show an increased risk of transformation and the risk increases as the degree of dysplasia increases [3].

Early diagnosis of oral cancer is critical to improve the survival rate of patients. The current strategies for screening of patients for oral premalignant and malignant lesions unfortunately miss a significant number of involved patients [4].

To date, the gold standard in the diagnosis of OPMDs is performed by clinical examination and histopathological evaluation, carried out by optical microscope observation of samples taken from patients through surgical biopsy, after staining with hematoxylin–eosin [5]. To improve the accuracy of clinical performances, Optical Coherence Tomography (OCT), can be used to acquire real time high resolution high-magnification images directly in vivo [6]. OCT is a label free and contact free diagnostic method of in vivo microscopy that makes use of non-ionizing radiation of white visible light, exploiting the optical properties of tissues [7]. The wavelengths produced by the light sources of OCT systems have a very wide range of lengths: it is visible white light tending towards infrared, therefore low frequencies, and wide wave lengths (center wave about 1000 nm). The use of a radiation with a fairly long wave length allows to penetrate deeper into the tissues [8]. Currently, the devices that can be used for in vivo investigations have a spatial resolution up to 1–2 µm for about 2 mm of depth, with a lateral width that varies according to the devices used: ranging from 2.5 mm to 10mmX10mm. These features allow to visualize the entire thickness of the epithelium and a large part of the connective surface [9].

The importance of epithelial thickness as a possible predictor of architectural changes was highlighted. A few of study provide that OCT is able to produce measurements that can compete with histopathological standards in a reproducible way, and also indicate how the epithelial thickness, combined with architectural changes, may lead to early differentiation of areas involved or free from cancer/dysplasia [7]. Furthermore, it is reported that architectural changes are statistically significant between benign / hyperplastic and/or hyperkeratotic lesions, lesions with dysplasia and healthy mucosa [10].

An ex vivo study based on an SS-OCT system that used optical attenuation model to identify and classify three types of tissues (normal mucosa, precancerous tissues, and oral cancer), showed how an appropriate algorithm can help to improve screening and the diagnosis of oral cancers [11].

As a non-invasive and real-time in vivo imaging technique, OCT can express identical information sufficient for oral cancer screening, but it has not been explored effectively for automatic diagnosis of oral cancer. However, to date the literature shows sufficient evidence regarding the peculiarities and similarities with traditional histopathology: in this way it is possible not always to resort to direct comparison with histological preparations and to avoid, if possible, surgical intervention on the patient [12].

The main purpose of this case series study is to evaluate in vivo discriminatory potential of OCT in a series of patients affected by OPMDs, before traditional biopsy, in order to support a preliminary evaluation by comparation with the site-specific healthy tissue.

## Materials and methods

### Patients selection

Patients with clinical appearances of OPMDs were recruited at Oral Medicine Section – CIR Dental School University of Turin, Italy. For each patient, data related to the clinical description were collected, followed by the OCT scan before bioptic sampling. Patients with the following clinical aspects are recruited: white lesions, white-red lesions, and ulcers. Patients with vegetating neoformations and/or with infiltrating characteristics, clinical appearance of Oral Squamous Cell Carcinoma (OSCC), non-cooperative patients, and patients whose sample was not suitable for OCT analysis (presence of artifacts) were excluded from the present work. All the lesions analysed, in addition to the clinical examination, were submitted for confirmation to investigative biopsy with histopathological diagnosis performed by Pathological Unit of Città della Salute e della Scienza di Torino. The present trial was conducted in line with the principles of the Helsinki Declaration of 1975, as revised in 2000 [13]; it was also accepted by the Research Board of the CIR-Dental School, University of Turin.

### OCT evaluation

**Fig. 1:**
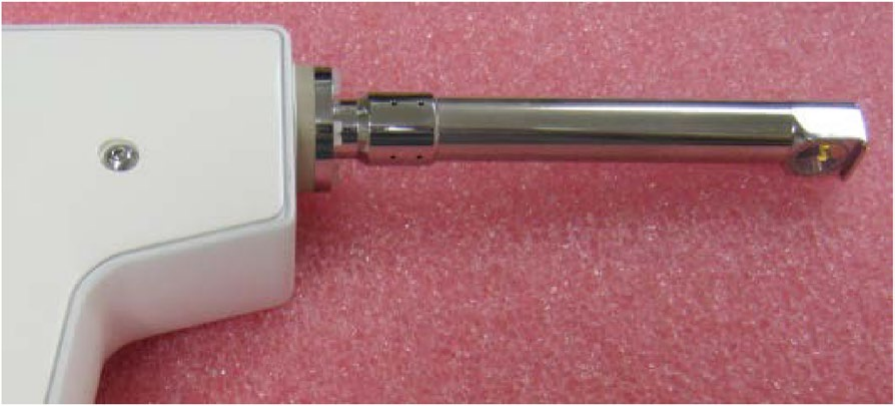
Prototype of manual probe for intraoral use

The latest variant of an OCT device, made for dermatological purposes (Swept Source OCT, by Vivosight® Michelson Diagnostics Ltd, version 2.0, Orpington, Kent, UK), was used (Fig. 1). The light source is provided by a Santec HSL-2000 laser with the following parameters: wavelength of 1305 +/- 15 nm, scanning field of 150 nm, axial and lateral resolution <10 μm, survey depth of 2 mm and 6 mm of image width. The same company has set up a prototype probe usable for intraoral examination (OCT endoscopic variant, version 2.1) of 124 mm in length, 15 mm in diameter and field of view with a maximum width of 6 mm^2^. This probe was installed on the motherboard (Fig. 1). In order to make it easier to use, contact with the mucosa to be examined is foreseen.

The in vivo OCT assessment of pathological tissue must compare with archive images of OCT scans of healthy tissue based on the anatomical site corresponding to the lesion. This is possible considering parameters as Keratinized Layer (KL), Epithelial Layer (EP), Basal Membrane (BM) and Lamina Propria (LP) and related evaluations (Table 1).

**Table 1:** OCT parameters for assessments of epithelial and subepithelial components

Parameter	Evaluation
**Keratin layer (KL)** Hyper-reflective area	Assessable/Not assessable
**Epithelial layer (EP)** Hypo-reflective area	Increase/Decrease/Not assesable
**Basal Membrane (BM)**	Assessable/Not Assessable
**Lamina propria (LP) **Hyper-reflective area	Increase/Decrease

This is a qualitative research study and adheres to the EQUATOR guidelines for research reporting using the Standards for Reporting Qualitative Research (SRQR) checklist for the collection and processing of instrumental and clinical data [14]. Given the exploratory nature of the study, no comparative assessment of the statistical difference between the categorical variables was performed. Preliminary measurements of the simple scans, in this phase of the study, are intended to illustrate and interpret a method that should be useful for identifying patterns of OPMDs. As regards the standard deviation of the measurements assumed as 0.05 mm, it refers to what was reported in a previous study [15]. In which it is highlighted that this value corresponds to an approximation of 50 microns susceptible to individual variation of the compartments of oral mucosa.

## Results

In the period between October 2021 and September 2022 it was recruited data of 11 patients (8 females a nd 3 males) (Table 2).

**Table 2:** Demographic characteristics of the sample

	TOTAL	MALES	FEMALES
PATIENTS (N)	11	3	8
AGE			
0–19		/	/
20–49		/	/
50–69		1	1
> = 70		2	7

The anatomical sites examined were tongue (divided into dorsum, border and ventral), gum, buccal mucosa. These areas were subjected to an observational survey (Table 3).

**Table 3:** Distribution by site

SITE	PATIENTS
Tongue Dorsum	1
Tongue Ventral Surface	2
Tongue Border Surface	3
Gingiva	2
Buccal Mucosa	3
**Total**	**11**

The elementary lesions observed in the 11 patients were classified into: white lesions, white and red lesions, ulcers. (Table 4).

**Table 4:** Elementary lesions distribution

	**TOT**	**MALES**	**FEMALES**
WHITE LESIONS	7	2	5
WHITE-RED LESIONS	1	1	/
ULCERS	3	/	3
**TOTAL**	**11**	**3**	**8**

The pathologies included in our OPMDs series were: Leukoplakia (LK), Oral Lichen Planus (OLP), Proliferative Verrucous Leukoplakia (PVL), Graft Versus Host Disease (GVHD), Micro invasive carcinoma (K-MICRO) (Table 5).

**Table 5:** Pathology distribution

	TOT	MALES	FEMALES
PVL	2	/	2
OLP	5	/	5
LEUKO	2	1	1
GVHD	1	1	/
K-MICRO	1	1	/
**TOTAL**	**11**	**3**	**8**

Although K-MICRO is not considered OPMDs, we decided to include it in this case-series to demonstrate that, whether in early stage cancer processes, OCT is useful in distinguishing between the two different ultrastructural patterns.

Below is the detailed description for each site examinated.

### Dorsum tongue

**Fig. 2:**
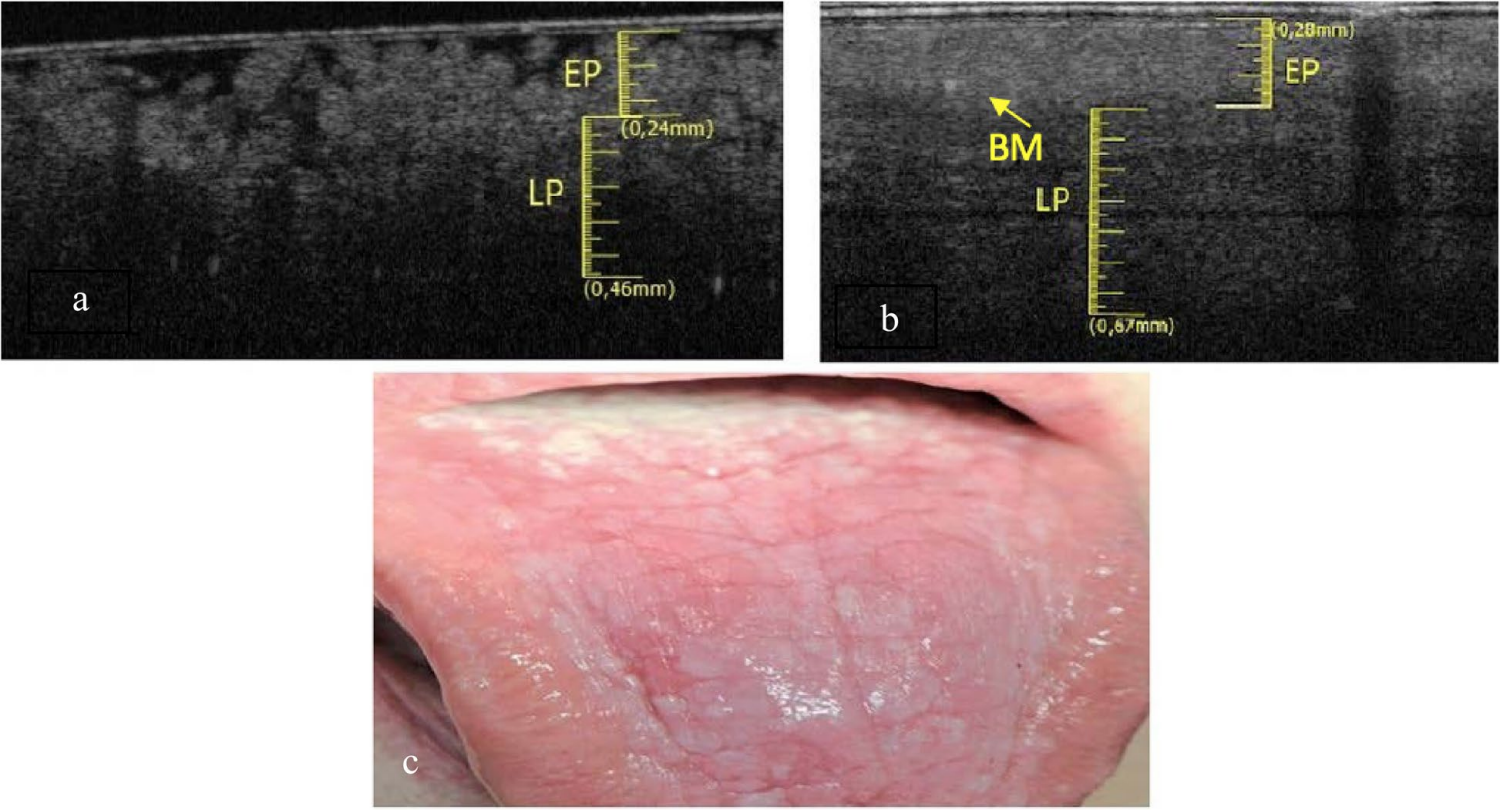
**a** Healthy OCT scan of dorsum of the tongue. **b** White lesion OCT scan of dorsum of the tongue. **c** Clinical image of withe lesion of dorsum of the tongue

The dorsum of the tongue was investigated in a 50-year-old male patient. The elementary lesion was a white lesion and the associated disease was Graft Versus Host Disease (GVHD) (Fig. 2c). OCT scan of healthy tissue shows that the characteristic digitations of the lamina propria towards the epithelial component are clear. It is not possible to distinguish the reflectance values related to the epithelial layer to the one related to the connective component. Therefore, it is possible to estimate the position of the BM, even if it is not clearly identifiable, due to the complex ultrastructural aspect of the site. (Fig. 2a). In Fig. 2b the epithelial layer is slightly increased (0.28 ± 0.05 mm) and shows a homogeneous surface, losing its “papillary” characteristic appearance. The reflectance tends to be increased in the epithelial structure. LP compart has increased its thickness (0.67 ± 0.05 mm).

The papillary introflexions of LP towards the epithelial layer are difficult to identify. The basal membrane appears to be assessable.

### Ventral tongue

The ventral area of the tongue was investigated in a subgroup of 2 female patients with a mean age of 77 years.

**Fig. 3:**
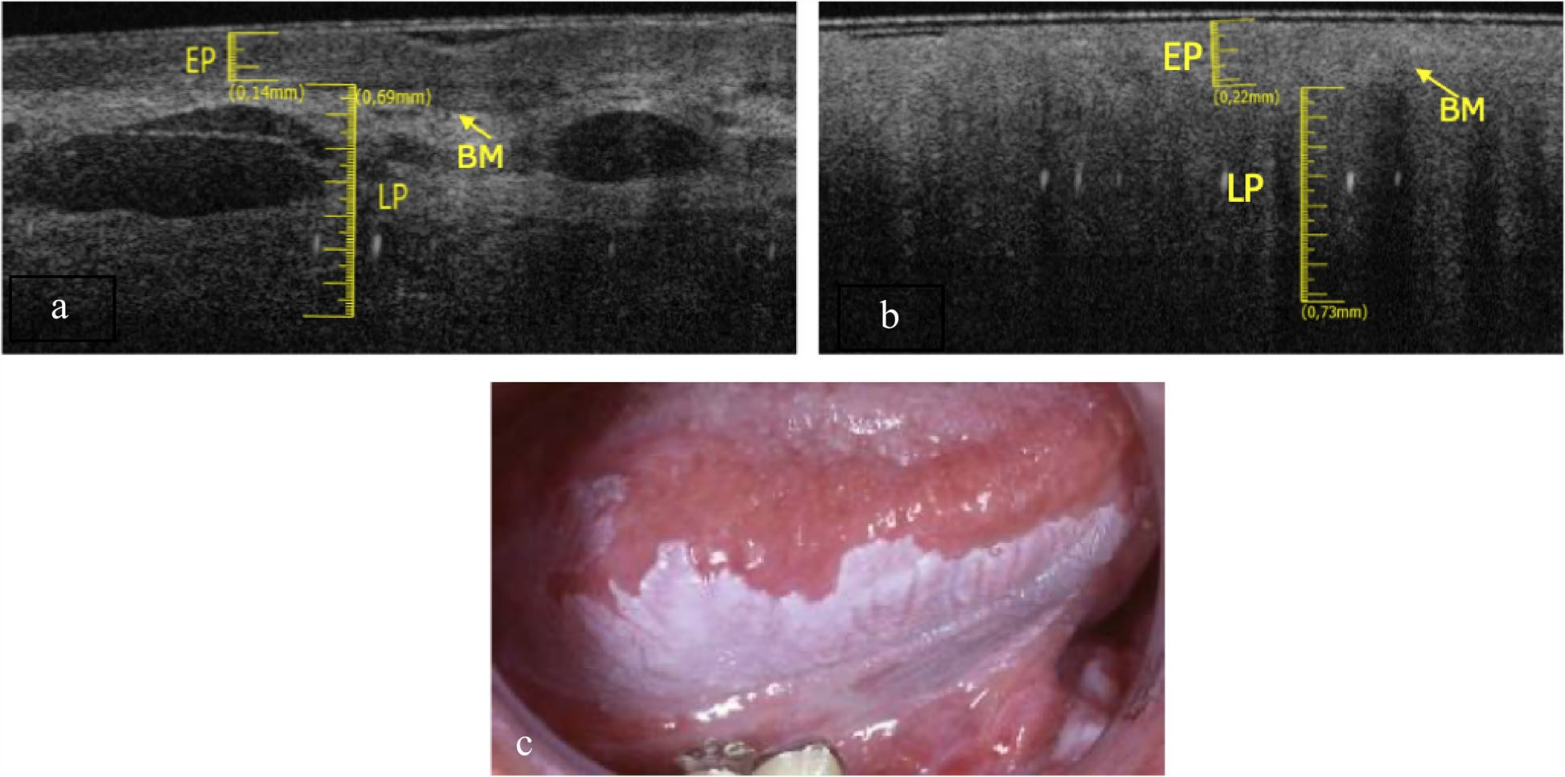
**a** Healthy OCT scan of ventral tongue surface. **b** Withe lesion OCT scan of ventral tongue surface. **c** Clinical image of withe lesion of ventral tongue surface

**Fig. 4:**
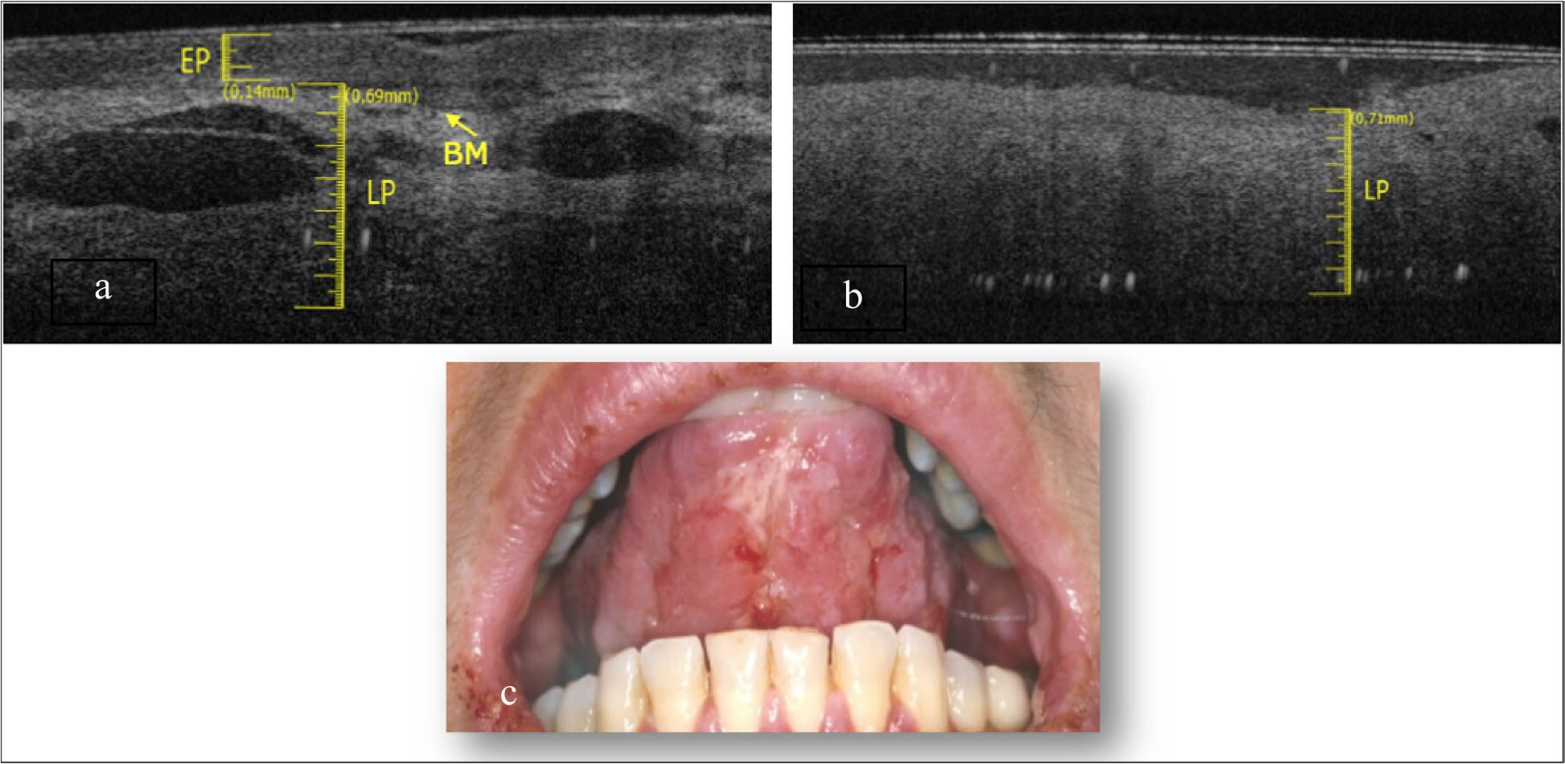
**a** Healthy OCT scan of ventral tongue surface. **b **Ulcer OCT scan of ventral tongue surface. **c** Clinical image of ulcer of ventral tongue surface

The elementary lesions found in this subgroup are a withe lesion and an ulcer. The pathology associated to white lesion was PVL (Fig. 3c) and the pathology associated to ulcer is OLP (Fig. 4c).

In the Fig. 3a the epithelial and connective components are clear, separated by a perfectly recognizable basal membrane. Between epithelium and connective tissue there is a clear and distinguishable gradient of reflectance. In the context of the lamina propria we can guess large horizontal “voids”, probably referable to the vascular structures.

The epithelial layer showed in Fig. 3b is increased (0.22 ± 0.05 mm), compared to the healthy counterpart (Fig. 3a) and appears more hyperreflective. The layer corresponding to the lamina propria has generally increased its thickness (0.73 ± 0.05 mm). It is still possible to estimate the position of the basal membrane. The large vascular structures present in the lamina propria are not so easily identifiable. The total thickness of epithelial layer and lamina propria is increased.

In the Fig. 4b it possible notice that LP is increased in thickness (0.71 ± 0.05 mm), whit a lower reflectance value than the healthy one (Fig. 4a). The EP and BM cannot be identified.

### Border tongue

**Fig. 5:**
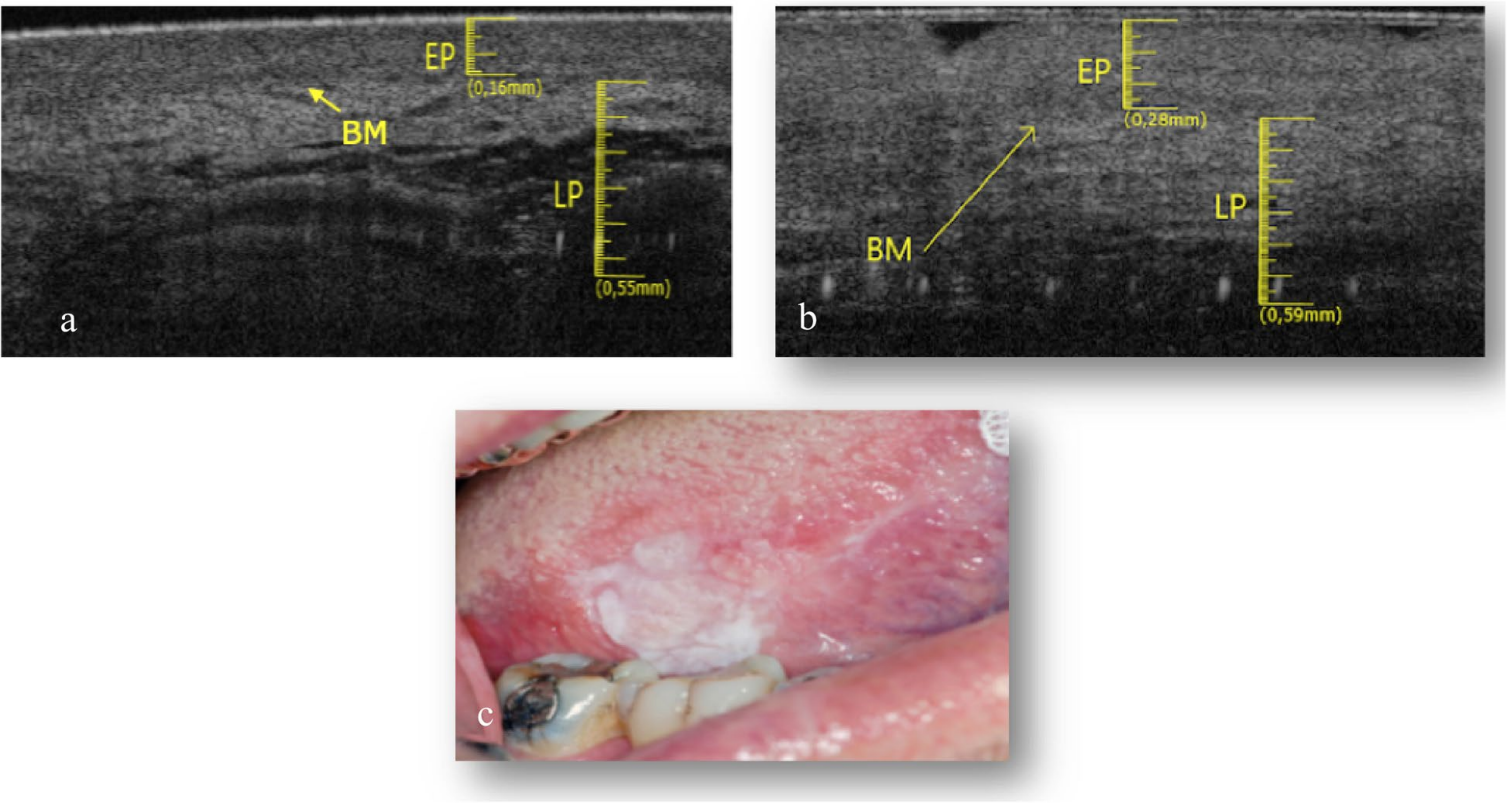
**a **Healthy OCT scan of tongue border surface. **b** White lesion OCT scan of border surface of tongue. **c** Clinical image of with lesion compatible with OLP of ventral surface of tongue

**Fig. 6:**
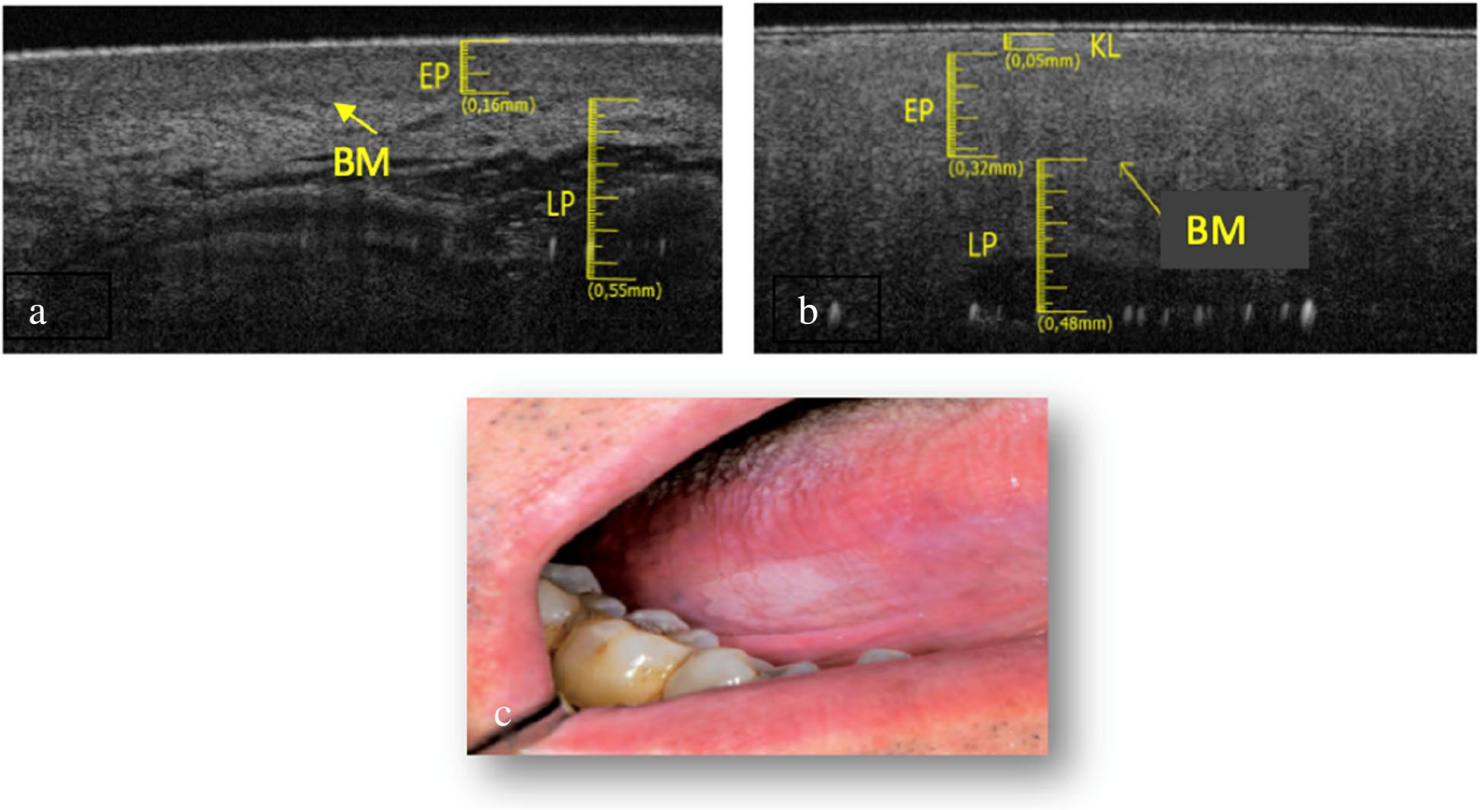
**a** Healthy OCT scan of tongue border surface. **b **White lesion OCT scan of of tongue border surface. **c** Clinical image of with lesion compatible with leukoplakia of tongue border surface

**Fig. 7:**
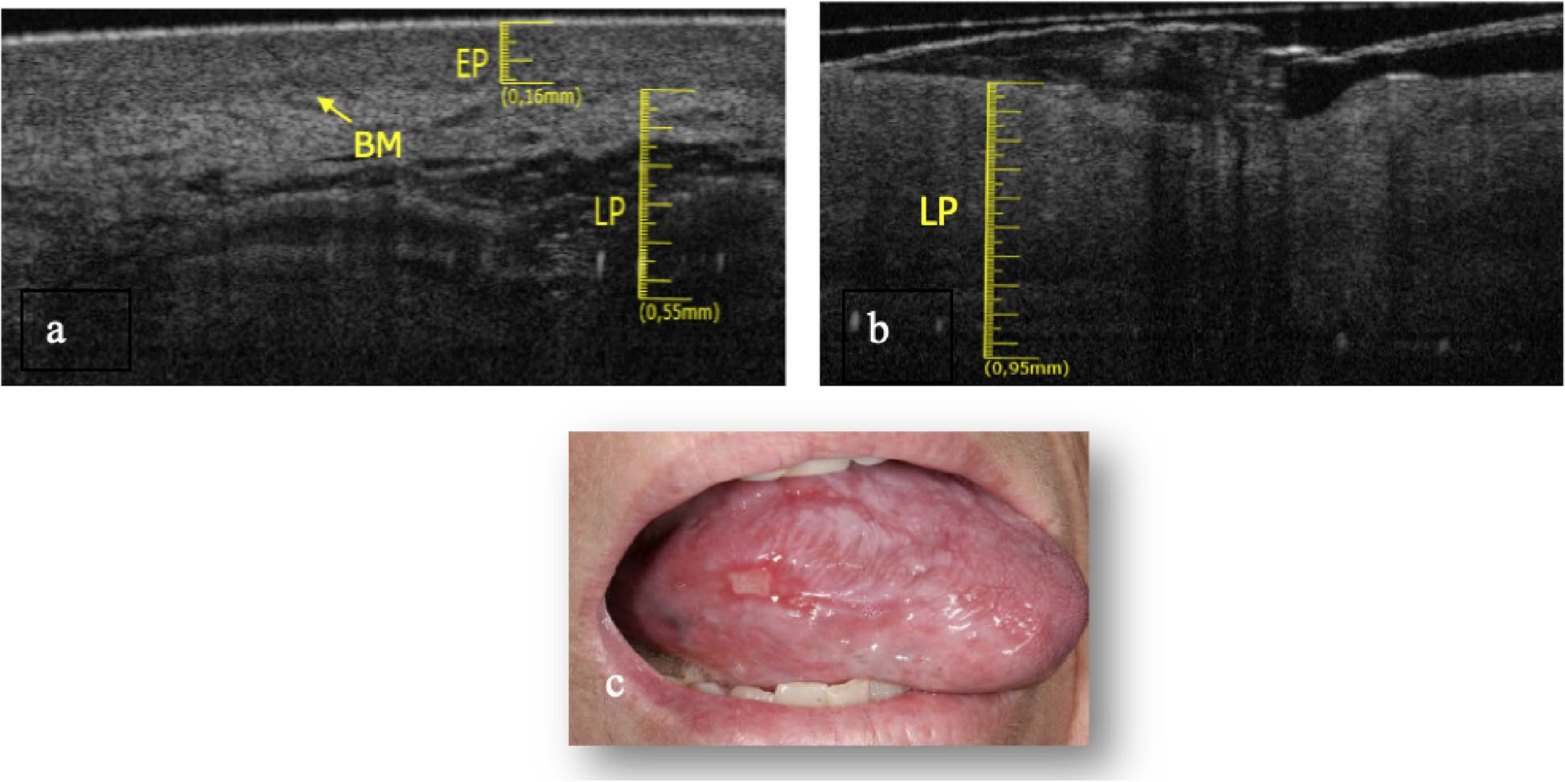
**a** Healthy OCT scan of tongue border surface. **b **Ulcer OCT scan of border surface of tongue. **c** Clinical image of ulcer of tongue border surface

The tongue border surface was investigated in a subgroup of 3 patients (1 M and 2 F) with an average age of about 73 years. The elementary lesions found in this subgroup are 2 withe lesions (Fig. 5c and Fig. 6c) and 1 ulcer (Fig. 7c). The associated pathologies for the withe lesions are 1 OLP and 1 LK. The associated pathology for the ulcer is OLP.

Compared to healthy tissue (Fig. 5a), in the Fig. 5b we can assess a thin keratin layer (0.05 ± 0.05 mm). EP appears slightly increased in thickness (0.32 ± 0.05 mm) as well as its reflectance. The least difference in reflectance between epithelial layer and the lamina propria makes it hard to assess the BM, even if its presence can be estimated in some points. Total thickness is increased referred to the healthy tissue.

The Fig. 6b shows evident hyper-reflectance of the EP layer concomitant with an increase in thickness (0.28 ± 0.05 mm) compared to healthy tissue (Fig. 6a). LP is increased (0.59 ± 0.05 mm). The position of the BM can still be clearly identifiable.

In Fig. 7b it is only assessable the LP layer, which is increased in thickness (0.95 ± 0.05 mm). Possible presence of a thin layer of fibrin could give rise to a superficial hyper-reflectance, while the underlying compart is hyporeflective. It is not possible to assess the BM. The total thickness increases, compared to the healthy one (Fig. 7a).

### Buccal mucosa

The buccal mucosa was investigated in a subgroup of 3 patients, all females, with an average e age of 70 years.

**Fig. 8:**
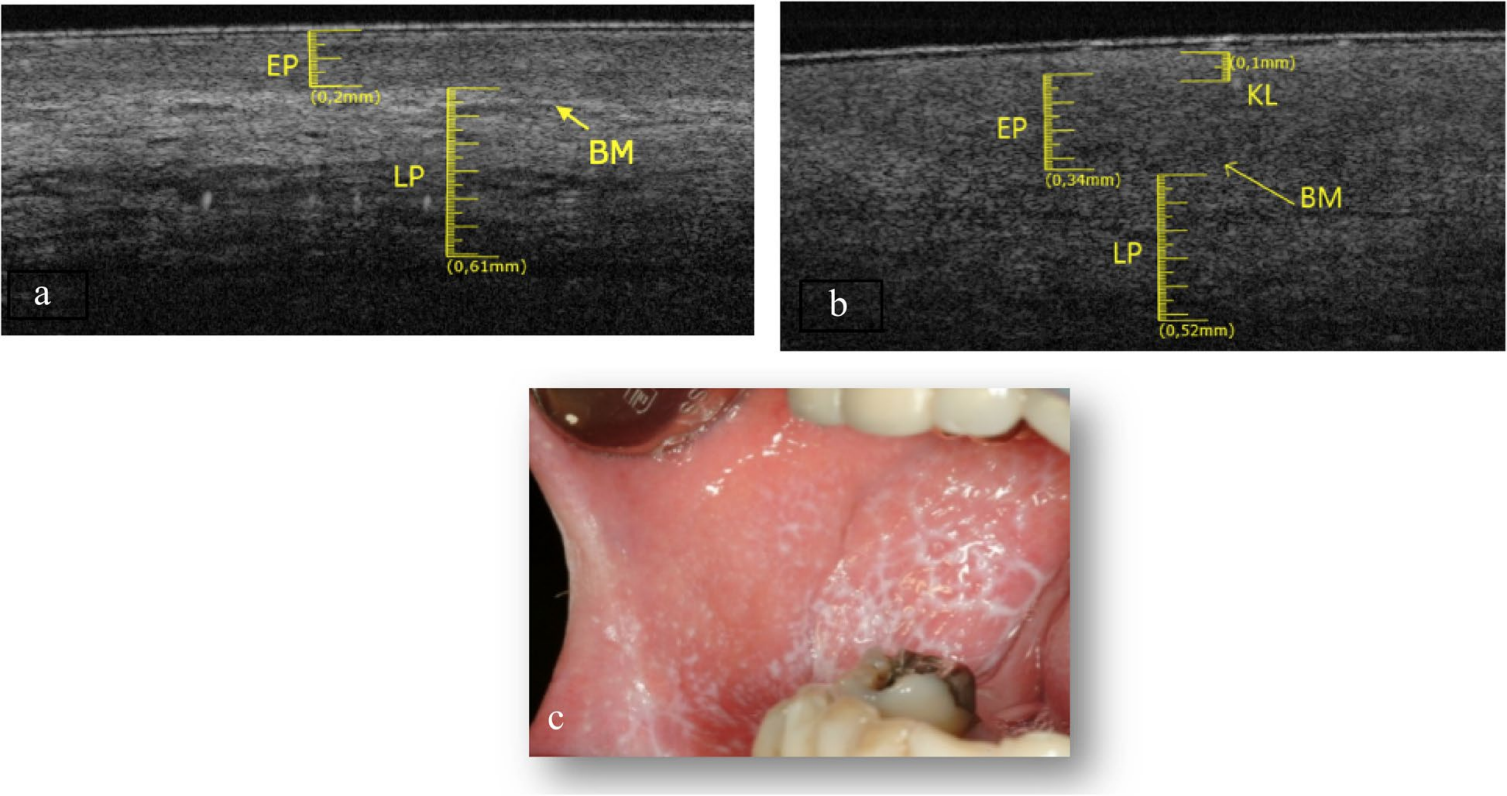
**a** Healthy OCT scan of buccal mucosa. **b** White lesion OCT scan of buccal mucosa. **c** Clinical image of withe lesion of buccal mucosa

**Fig. 9:**
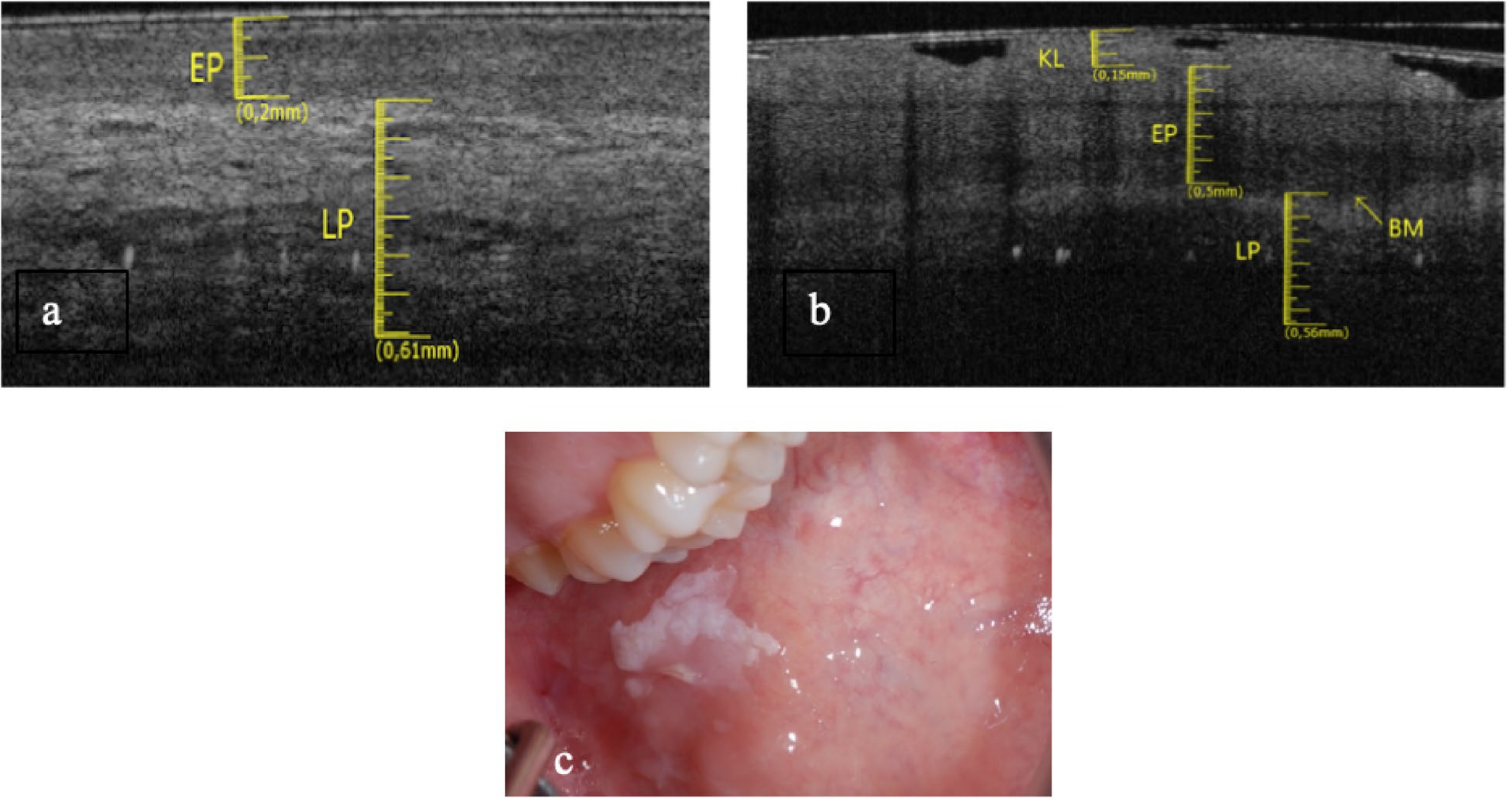
**a** Healthy OCT scan of buccal mucosa. **b** White lesion OCT scan of buccal mucosa. **c** Clinical image of withe lesion of buccal mucosa

**Fig. 10:**
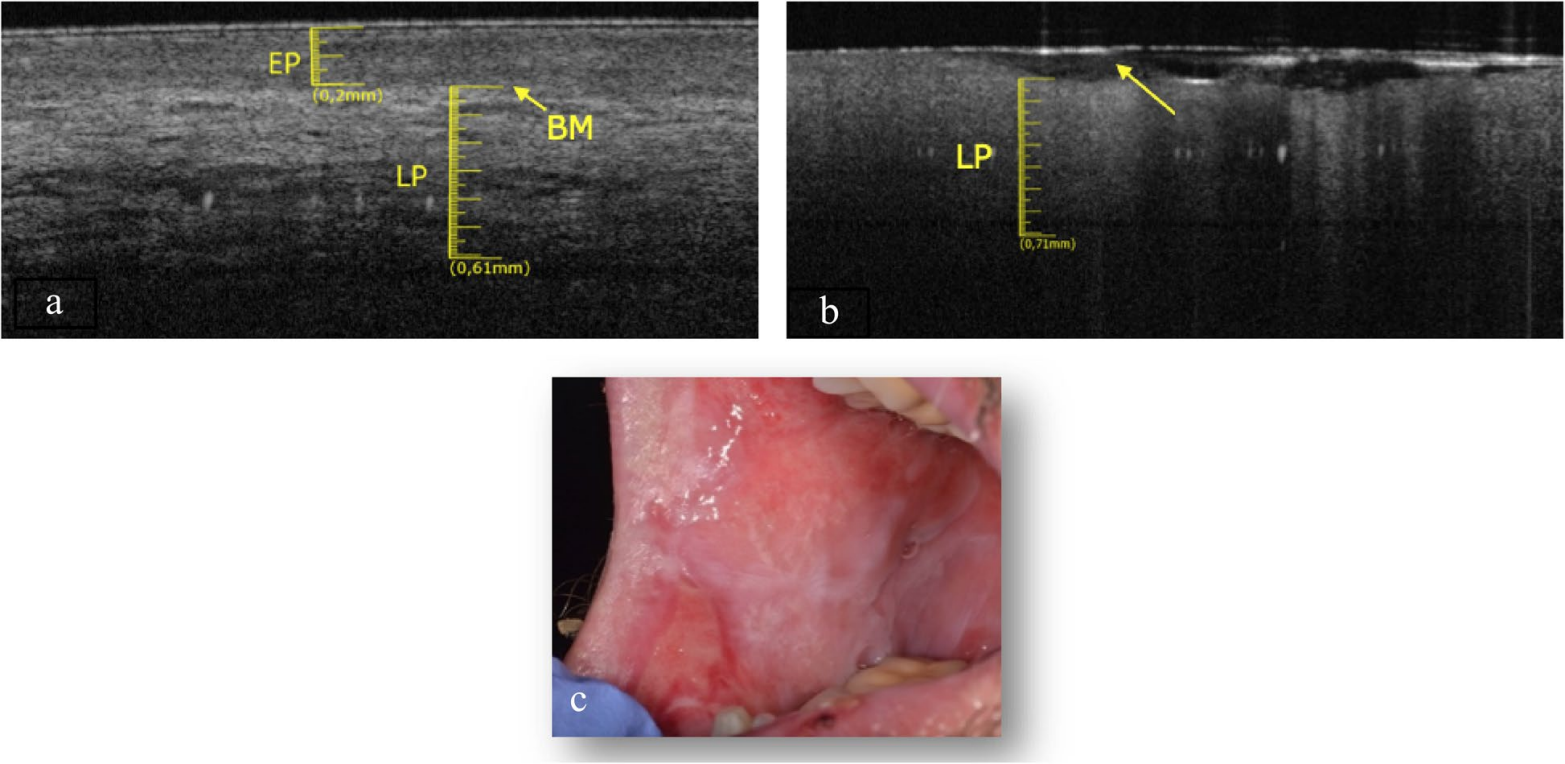
**a** Healthy OCT scan of buccal mucosa. **b** Ulcer OCT scan of buccal mucosa. **c** Clinical image of ulcer of buccal mucosa

The elementary lesions found in this subgroup are 2 white lesions (Fig. 8c and Fig. 9c) and 1 ulcer (Fig. 10c). The pathologies associated with withe lesions are 1 OLP and 1 LK. The pathology associated with the ulcer is 1 OLP.

In the Fig. 8b is possible to assess the presence of a thin KL (0.1 ± 0.05 mm), the EP appears increased in thickness (0.34 ± 0.05 mm) compared to healthy tissue (Fig. 8a). The LP layer appears decreased in thickness (0.52 ± 0.05 mm) as well as in reflectance. The BM can still be assessable. Total tissue thickness is increased.

In the Fig. 9b an irregular EP surface and increased KL (0.15 ± 0.05 mm) compared to the healthy one (Fig. 9a) is appreciated. The LP layer is decreased (0.56 ± 0.05 mm) and it has a lower reflectance value than the healthy one. The basal membrane is determinable and appears intact. Total tissue thickness is increased.

The Fig. 10b shows an LP layer detectable, increased in thickness (0.71 ± 0.05 mm) and with low reflectance value, respect the healthy one (Fig. 10a). It is not possible to assess the BM. Total tissues thickness is decreased.

Gingiva.

The adherent gingiva was investigated in a subgroup of 2 patients, a male and a female, with a n average age of 76 years.

**Fig. 11:**
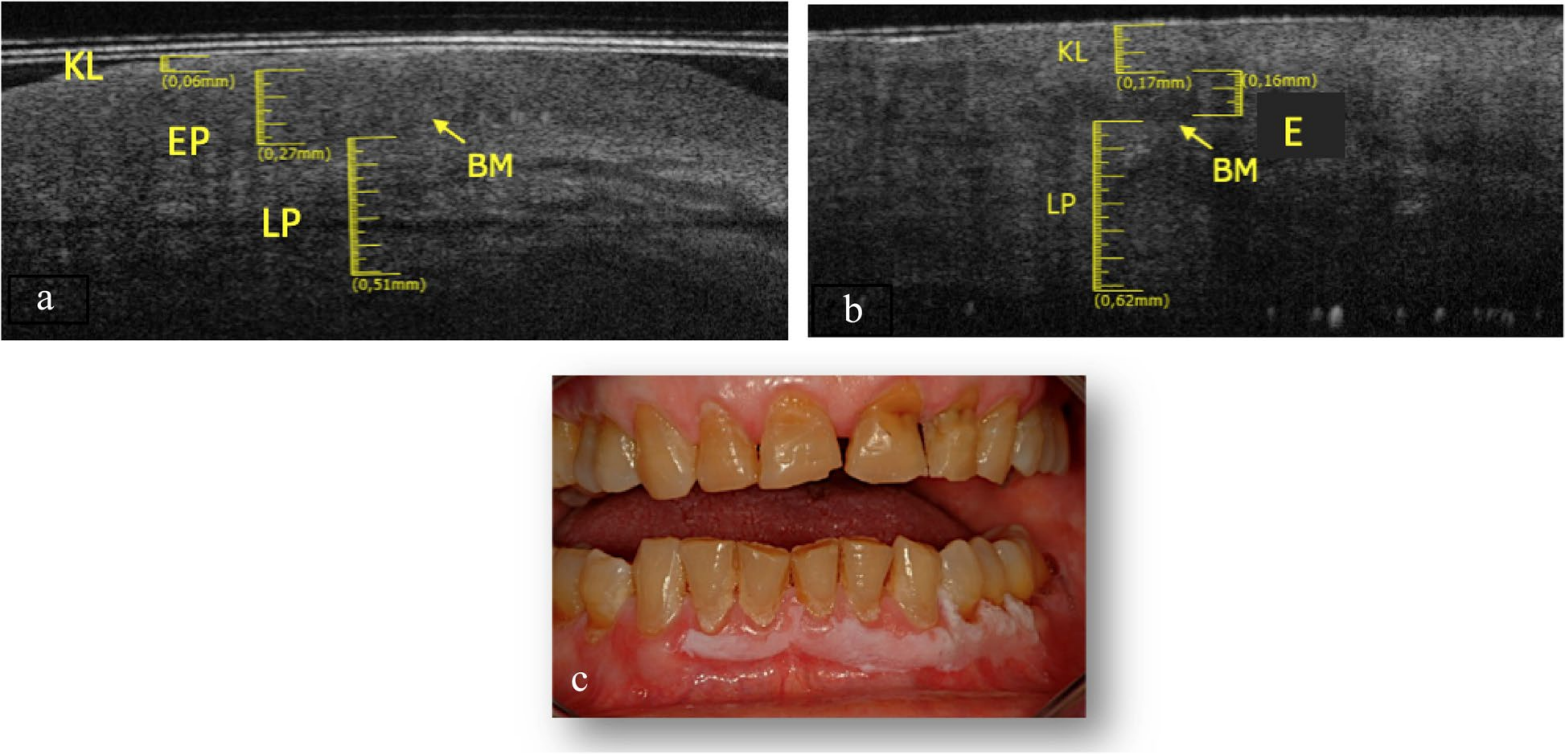
**a** Healthy OCT scan of adherent gingiva. **b** White lesion OCT scan of adherent gingiva. **c** Clinical image of withe lesion of adherent gingiva

**Fig. 12:**
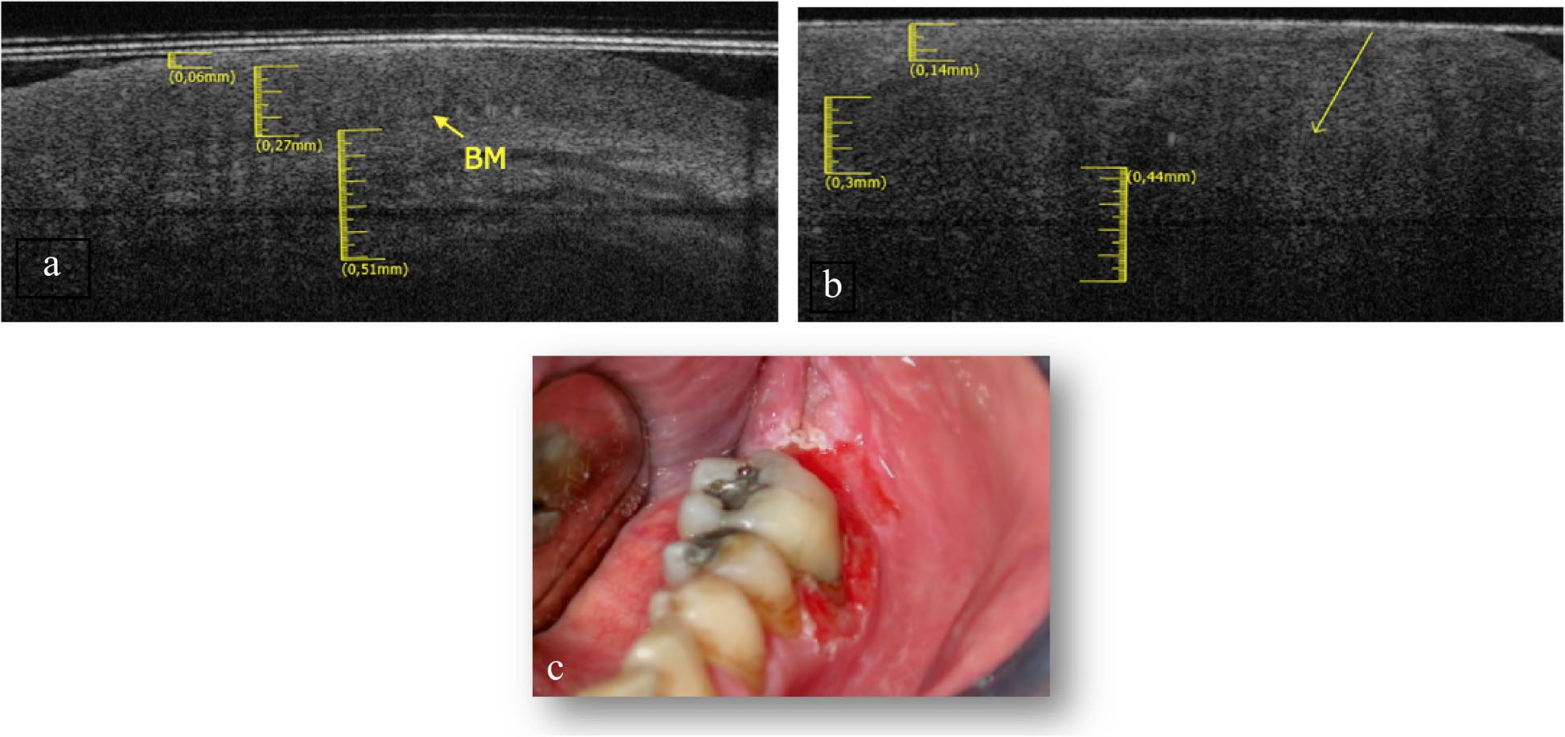
**a** Healthy OCT scan of adherent gingiva. **b** White and red lesion OCT scan of adherent gingiva. **c** Clinical image of withe and red lesion of adherent gingiva

The elementary lesions found in this subgroup are a white lesion (Fig. 11c)and a white-red lesion (Fig. 12c). The pathology associated with withe lesion is PVL and the pathology associated with the red and withe lesion is Microinvasive Carcinoma (K MICRO).

The keratin layer in the Fig. 11b is increased (0.17 ± 0.05 mm) compared to the healthy counterpart (Fig. 11a). The EP layer is decreased (0.16 ± 0.05 mm). LP is increased in thickness (0.62 ± 0.05 mm). It is still possible to estimate the position of the BM. Total tissue thickness is increased.

In Fig. 12b KL is increased (0.14 ± 0.05 mm) compared to the healthy counterpart (Fig. 12a). EP layer is increased (0.3 ± 0.05 mm). LP appears decreased in thickness (0.44 ± 0.05 mm). The arrow shows vertical “digitations” in the thickness of LP (which does not occur in the healthy tissue): a sign of tissue disruption. Consequently BM is no longer identifiable. Total tissue thickness is increased.

Table 6 shows the relationship between healthy tissue, elementary lesion related to the different pathologies and difference in the thickness values of the epithelial layer (EP) connective layer (LP) presence of basement membrane (BM) and keratin layer (KL) in different oral mucosa sites.

**Table 6:** Values of the thicknesses of the epithelial and connective layer of the different lesions related to singular pathologies and comparing with healthy tissues

Site	Healthy Tissues	Clinical Lesion	Pathology	Keratin Layer (KL)	Epithelial Layer (EP)	Basal Membrane (BM)	Lamina Propria (LP)
Dorsum Tongue	**EP**: 0.24 ± 0.05 mm **LP**: 0.46 ± 0.05 mm	**White Lesion**	GVHD	Not Assessable	Increased 0.28 ± 0.05 mm	Assessable	Increased 0.67 ± 0.05 mm
Ventral Tongue	**EP**: 0.14 ± 0.05 mm **LP**: 0.69 ± 0.05 mm	**White lesions**	PVL	Not Assessable	Increased 0.22 ± 0.05 mm	Assessable	Increased 0.73 ± 0.05 mm
		**Ulcer**	OLP	Not Assessable	Not Assessable	Not Assessable	Increased 0.71 ± 0.05 mm
Border Tongue	**EP**: 0.16 ± 0.05 mm **LP**: 0.55 ± 0.05 mm	**White Lesion**	OLP	Assessable 0.05 ± 0.05 mm	Increased 0.32 ± 0.05 mm	Assessable	Decreased 0.48 ± 0.05 mm
		**White Lesion**	LK	Not Assessable	Increased 0.28 ± 0.05 mm	Assessable	Increased 0.59 ± 0.05 mm
		**Ulcer**	OLP	Not Assessable	Not Assessable	Not Assessable	Increased 0.95 ± 0.05 mm
Buccal Mucosa	**EP**: 0.2 ± 0.05 mm **LP**: 0.61 ± 0.05 mm	**White Lesion**	OLP	Assessable 0.1 ± 0.05 mm	Increased 0.34 ± 0.05 mm	Assessable	Decreased 0.52 ± 0.05 mm
		**White Lesion**	LK	Assessable 0.15 ± 0.05 mm	Increased 0.5 ± 0.05 mm	Assessable	Decreased 0,56 ± 0.05 mm
		**Ulcer**	OLP	Not Assessable	Not Assessable	Not Assessable	Increased 0.71 ± 0.05 mm
Gingiva	KL:0.06 ± 0.05 mm EP: 0.27 ± 0.05 mm LP: 0.51 ± 0.05 mm	**White Lesions**	PVL	Increased 0.17 ± 0.05 mm	Decreased 0.16 ± 0.05 mm	Assessable	Increased 0.62 ± 0.05 mm
		**White-Red Lesion**	K-MICRO	Increased 0.14 ± 0.05 mm	Increased 0.3 ± 0.05	Not Assessable	Decreased 0.44 ± 0.05 mm

In white lesions total thickness could decrease: lesions could appear white because there is an increase of the keratin component even if matched with a decreasing of the total epithelial – connective thickness. In this case series, the pathology that most often creates a decrease in thickness is OLP.

OLP can also manifest with atrophic-erosive lesions but at the same time, it can also give white lesions with a decrease of the total thickness in the face of an increase in the keratin component.

## Discussion

The ability of OCT to distinguish between precancerous lesions and oral cancer is still unclear. A recent study calculated a sensitivity (93–96%) and specificity (74–49%) in ex-vivo OSCC, leukoplakia with hyperplasia and healthy simple tissue by creating computational models of these tissues and employing an artificial neural network analysis algorithm [16].

Another recent meta-analysis reported the trend of OCT in OPMDs to be less sensitive (0.8960) and specific (0.8962) than OSCC (0.9316) [17].

The possible explanation for this result lies in the absence of systematic reviews that analyzed only the OCT patterns only for OPMDs, in the lack of standardization of instrument settings and image measurement methods for the universal use of data. However, the generally high sensitivity and specificity indicate the potential utility of OCT for OPMD follow-up.

The use of artificial intelligence will be the real upgrade of use of OCT in Oral Medicine [16]. Hence, there is the need for further research to establish unambiguous criteria for the classification of in vivo patterns of OMPDs.

Currently, the main limitations of this procedure depend on both the device and the operator. The first concerns the device itself, i.e. the limited diffusion of OCT in the dental field with high-performance intraoral OCT handheld probes and the limited imaging depth [18]. The second is a bias that concerns the single operator: the reading of the images and their interpretation involve a simple learning curve, but it is always the direct observation of one or a few specialists [19]. It is desirable to compare the measurements and images with an expert team across a larger case series and more accurately determine the different site-specific pathological patterns to reduce this bias.

Knowledge of OCT healthy tissue is essential for understanding ultrastructural alterations [20].

In the healthy tissue, the EP layer is always hypo-reflective (with the exception of the superficial KL, if it is present), the LP is always hyper-reflective with an internal non-reflective areas; the difference between these two degrees of reflectance leads to estimate the position of the BM.

The data of this study are broadly in agreement with the study of Albrecht et al. [21] which claims the thickness of the EP 0.12 ± 0.015 mm, within the range of the average measurements obtained in our scans measured of 0.14 mm.

Furthermore, the authors affirm that it is possible to identify large vascular structures in the structure of the lamina propria: also, this data is in agreement with our evidence. The same author argues that it is difficult to highlight the basal membrane in the region of the ventral tongue, while we were able to clearly identify it. This could be due to the different position of the intraoral probe: in our study the probe was brought into contact with the mucosa.

According to some authors, the hyper-reflectance of the epithelial layer can be understood as a good parameter to differentiate diseased tissues from healthy ones [22, 23]. It must be borne in mind that even some benign lesions could have hyperkeratosis aspects: this can lead to increased reflectance. Therefore, the epithelial hyper-reflectance must always be correlated to other structural characteristics, in order to take it into consideration as a diagnostic parameter. Considering the aforementioned, it becomes important trying to identify the BM, bearing in mind the overexposed difficulties in finding it (especially for the buccal mucosa and adherent gingiva).

The site-specificity in which the lesions appear is the first step in being able to identify the pathological tissue from the healthy one.

Panzarella et al. [24] coded the different sites of the oral cavity where OSCC lesions occurred. On the basis of this study to intercept architectural changes indicative of a given disease is evident in the analysis of the contiguous t issues from healthy to pathological sites turns out to be the starting point for placing the peculiarities of the KL,EP, BM and LP layers, their presence or absence and their thickness. This last feature seems to be the one that can most easily relate the lesion to a pathology.

The same authors highlight how in dorsum tongue there is no real difference in reflectance that allows us to distinguish KL from EP, therefore the anatomy is not the same as other structures that appear flat.

Although the investigated sites are dissimilar, the common aspects of all the OPMDs analyzed were two: increase in epithelial reflectance and decrease in reflectance of the lamina propria [25]. The increase in the reflectance of the EP layer can be a direct consequence of cellular hyper-proliferation: high cellularity lead to have a different patterns of the pathological EP layer compared to the healthy tissue [26].

For this reason, ulcers patterns could highlight a superficial hyper-reflectance because epithelial cells increase proliferation starting from the edges of the lesion to try to re-epithelialize the area. In extensive ulcers also the central fibrin panniculus could result in greater reflectance, but this data should be further investigated. The presence of hyper-reflectance area in the residual epithelium of an OLP ulcer is due to a mild hyper-keratosis or hyper-parakeratosis but the loss of the clear difference between the two reflectance degrees (between epithelial and connective tissue) makes it difficult to identify the basal membrane [15]. To date is difficult to establish whether the inability to distinguish the BM is due to the anatomical characteristics of sites or to diseases. However, the evolution of the carcinogenesis process makes more difficult to identify the epithelial and connective layers and consequently the BM [7, 24]. The limit of the study remains the subjective interpretation of the operator who can request help from objective measurements thanks to the information provided by the device.

It is almost established that the more the lesions evolve towards malignity, the less the layers and the basal membrane are identifiable [7, 27]. The determinability of an epithelial thickness cannot be assumed, alone, as a predictor of malignancy, but must always be correlated with the other structural aspects, (increased thicknesses of the epithelial layer can also be found in benign lesions) [28]. The lamina propria, it is assumed, loses in reflectance because there is an overlap of the inflammatory component (granulocytic exocytosis, edema, etc.). Even when this is increased in thickness in OCT, it is not the connective component that has grown, but the quantity of the inflammatory infiltrate: there is a decrease in the density of the lamina propria and, therefore, a decrease in its degree of reflectance. This is a typical finding of immune-mediated diseases [29–31].

The loss of the clear difference between the two reflectance degrees (between epithelial and connective tissue) makes it difficult to identify the basal membrane: the lesions for which it was more difficult to identify the basal membrane were those affecting the buccal mucosa, the adherent gingiva, the border and the ventral tongue.

Specifically, it is difficult to identify the basal membrane in: OLP ulcers on the edge of the tongue, ventral tongue and buccal mucosa, spot on the buccal mucosa due to OLP, inhomogeneous white/red lesion on adherent gingiva (K-MICRO) and PVL on adherent gingiva. From the analysis of our images it was possible to notice a characteristic aspect of OLP ulcers: the almost total loss of the epithelial component and a uniform LP hypo-reflectivity of the connective component. This makes it very difficult to estimate the position of the basal membrane. In trauma ulcers, this specific pattern appears not to occur so frequently [32] probably because there is an attempt to repair the lesion by the epithelial cells, coming from the surrounding edges, which undergo an active proliferation. It has been shown that high cellularity and cells with very active mitochondria lead to a different image of the epithelial layer compared to the healthy one [26]. A preliminary work [33] highlights how there may be a component of hyper-reflectance in the residual epithelium of an OLP ulcer due to a mild hyper-keratosis or hyper-parakeratosis.

A white lesion does not always clinically correspond to an increased epithelial tissue thickness. Even if we are considering keratinized lesions, it is not always possible to assess a KL, because often the keratinization process involves the entire thickness of EP and not only the superficial layer. For this reason, withe lesions on the tissue surface can lead to a decrease in the depth of light penetration, effectively limiting the potential of the OCT system.

We conducted our in vivo investigation on different types of lesions without preliminarily knowing the result of the initial histopathological analysis. In our series, an inhomogeneous white-red lesion turned out to be a microinvasive carcinoma and the OCT pattern already describes the typical peculiarities of invasive oral cancer such as the non-determinability of the basement membrane [32]. Despite we are still able to distinguish thicknesses similar to the typical patterns of the OPMDs described above.

Although this study did not investigate the possibility of recognizing the presence of dysplasia using the OCT system, it is suggested that dysplasia is also present in perilesional clinically healthy tissue [4, 34]. It is good to keep this aspect in mind when investigating oral lesions in OCT, in order to correctly interpret suspect images in perilesional mucosa.

## Conclusions

To the best of our knowledge this is one of the few studies investigating the in vivo use of OCT in OPMDs.

In vivo use of this device by the generalist physician, with adequate training, will improve its diagnostic performances during the long-term follow-up of the patient with potential malignant disorder reserving the biopsy with histopathological traditionally examination if a worsening or malignant evolution pattern is observed.

The Oral Medicine specialist should use in vivo-OCT not only for short-medium follow-up for patients most at risk of malignant evolution but also to monitor outcomes of medical or surgical therapy and any recurrences of OPMDs at a non-invasive and ultrastructural level.

Further studies with large sample series will be needed to validate the precise values of the thicknesses of different epithelial layers during OCT evaluation to obtain more significant results with reference to the specific sites of the various districts of the oral mucosa.

## Data Availability

The datasets used and analysed during the current study are available from the corresponding author on reasonable request*.*

## Abbreviations

OCT: Opical coherence Tomografy
OPMD: Oral Potential Malignant Disorder
KL: Keratin Layer
EP: Epithelial Layer
LP: Lamina Propria
BM: Basal Membrane
OSCC: Oral Squamosu Cell Carcinoma
LK: Leuplakia
OLP: Oral Lichen Planus
PVL: Proloferative Verrucuse Leucoplakia
Kmicro: Mircoinvasive Carcinoma
