# Correction: Potential use of optical coherence tomography in oral potentially malignant disorders: in-vivo case series study

**DOI:** 10.1186/s12903-023-03523-9

**Published:** 2023-11-07

**Authors:** Alessio Gambino, Eugenio Martina, Vera Panzarella, Tiziana Ruggiero, Giorgia El Haddad, Roberto Broccoletti, Paolo G. Arduino

**Affiliations:** 1 Department of Surgical Sciences, CIR Dental School, University of Turin, Via Nizzan. 230, 10123 Turin, Italy; 2 Department of Surgical, Oncological and Oral Sciences, University of Palermo, Palermo, Italy

Correction: BMC Oral Health (2023) 23:540

10.1186/s12903-023-03263-w

In this article [1], the wrong figure appeared as Fig. 6; the correct Fig. 6 should have appeared as shown below. Also, the TRN was included in the article in error and has been removed from the original article.

**Fig. 6:**
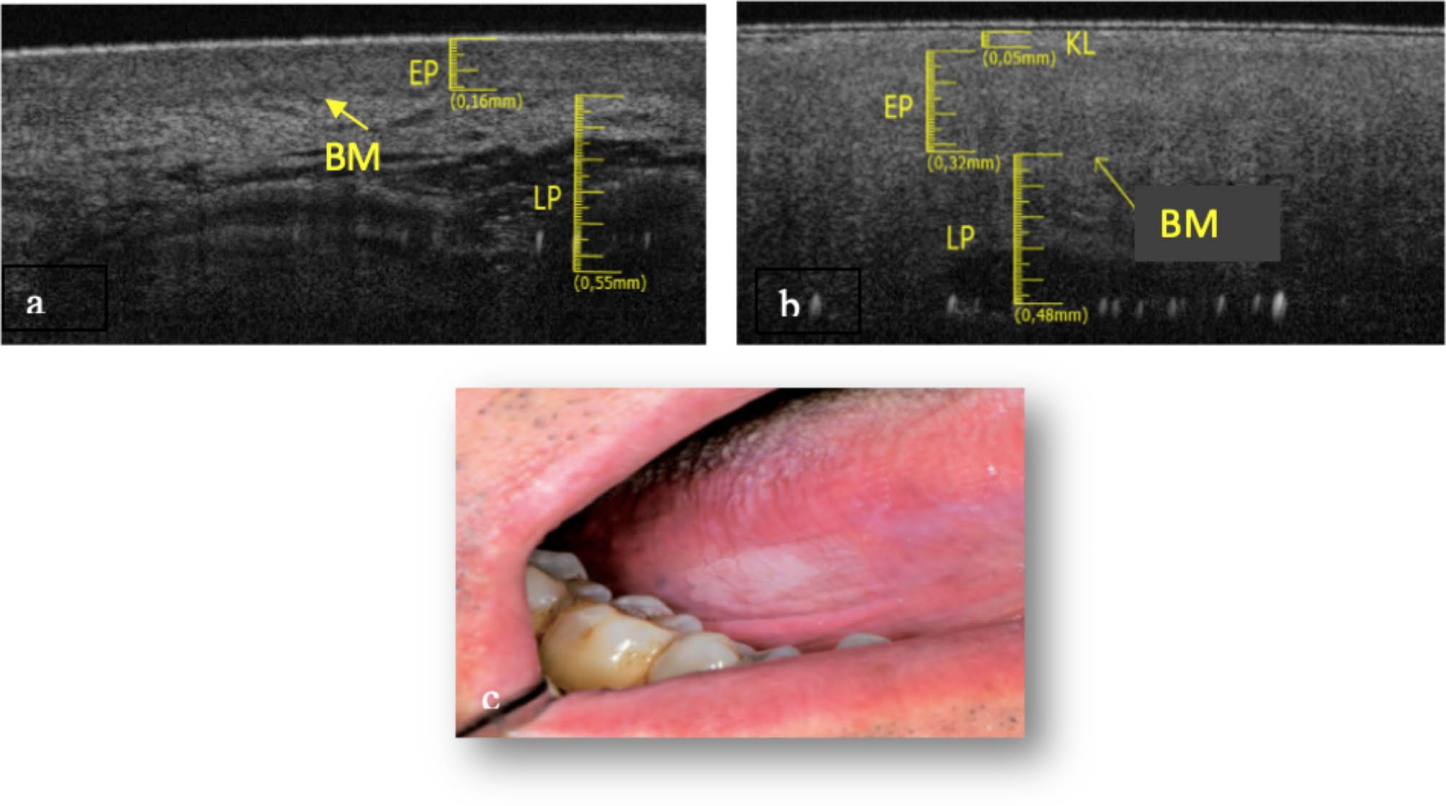
**a** Healthy OCT scan of tongue border surface. **b** White lesion OCT scan of tongue border surface. **c** Clinical image of white lesion compatible with leukoplakia of tongue border surface

The original article has been corrected.
